# Two-UAV Intersection Localization System Based on the Airborne Optoelectronic Platform

**DOI:** 10.3390/s17010098

**Published:** 2017-01-06

**Authors:** Guanbing Bai, Jinghong Liu, Yueming Song, Yujia Zuo

**Affiliations:** 1 Chinese Academy of Science, Changchun Institute of Optics Fine Mechanics and Physics, Key Laboratory of Airborne Optical Imaging and Measurement, #3888 Dongnanhu Road, Changchun 130033, China; baigb@ciomp.ac.cn (G.B.); himyf0319@126.com (Y.S.); mzyj0617@126.com (Y.Z.); 2 University of Chinese Academy of Sciences, #19 Yuquan Road, Beijing 100049, China

**Keywords:** UAV (unmanned aerial vehicles), airborne optoelectronic platform, intersection localization, coordinate transformation, accuracy analysis, adaptive Kalman filtering

## Abstract

To address the limitation of the existing UAV (unmanned aerial vehicles) photoelectric localization method used for moving objects, this paper proposes an improved two-UAV intersection localization system based on airborne optoelectronic platforms by using the crossed-angle localization method of photoelectric theodolites for reference. This paper introduces the makeup and operating principle of intersection localization system, creates auxiliary coordinate systems, transforms the LOS (line of sight, from the UAV to the target) vectors into homogeneous coordinates, and establishes a two-UAV intersection localization model. In this paper, the influence of the positional relationship between UAVs and the target on localization accuracy has been studied in detail to obtain an ideal measuring position and the optimal localization position where the optimal intersection angle is 72.6318°. The result shows that, given the optimal position, the localization root mean square error (RMS) will be 25.0235 m when the target is 5 km away from UAV baselines. Finally, the influence of modified adaptive Kalman filtering on localization results is analyzed, and an appropriate filtering model is established to reduce the localization RMS error to 15.7983 m. Finally, An outfield experiment was carried out and obtained the optimal results: $\sigma_{B}=1.63\times 10^{-4}\;({}^{\circ})$ , $\sigma_{L}=1.35\times 10^{-4}\;({}^{\circ})$ , $\sigma_{H}=15.8\;(m)$ , $\sigma_{sum}=27.6\;(m)$ , where $\sigma_{B}$ represents the longitude error, $\sigma_{L}$ represents the latitude error, $\sigma_{H}$ represents the altitude error, and $\sigma_{sum}$ represents the error radius.

## 1. Introduction

As an important tool for localization, photoelectric measuring equipment is playing an important role in military and civilian applications [1,2,3,4]. According to the base variety, modern photoelectric measuring equipment is mainly divided into ground-based photoelectric theodolites, surveying vessels and airborne photoelectric platforms. However, when being used in actual reconnaissance and localization, photoelectric theodolites and surveying vessels are often affected by operating range and other factors so that they cannot track and locate the targets all of the way. In this context, owing to the high maneuverability of UAVs, airborne photoelectric platforms are playing a more and more important role in reconnaissance and localization [5,6,7,8,9]. In recent years, with rapid development of UAV (unmanned aerial vehicles) technology, UAV-borne optoelectronic positioning devices have found wider and wider use in reconnaissance and monitoring, and have received more and more researchers’ attention [10,11,12,13]. Traditionally, an airborne photoelectric platform works in a single-station REA (range, pitch, and azimuth) localization manner, that is, to locate the target by using the distance *R* (of the target in relation to the platform), pitch angle *E*, and azimuth angle *A*, measured by the platform [14], as well as the position and attitude measured by airborne GPS (Global Positioning System)/INS (inertial navigation system). However, this method has a limited positioning accuracy, and as the laser range finder (LRF) it relies on has a limited operating range, this method has a narrower use. Therefore, a new localization method is needed to meet the requirement of high-precision localization. 

Hosseinpoor et al. [15,16] used a UAV with RTK (real-time kinematic)-GPS for estimation and localization, and processed the localization results through extended Kalman filtering. Their method is characterized by smooth localization results, simple and achievable equipment, but with limited positioning accuracy. Conte et al. [17] achieved the target localization through a micro aerial vehicle (MAV), a vehicle applicable to ground targets within a short range. Frew [18] located the ground targets through two-UAV cooperative localization. Ross et al. [19] identified and located the ground targets through a real-time algorithm and demonstrated, through testing, a significant influence of GPS accuracy on localization accuracy. Sohn et al. [20] proposed the use of triangulation for target localization. Cheng et al. [21] located a static ground target through two-point intersection localization, and proposed the use of least-squares iteration to improve the localization accuracy. Sharma et al. [22] located moving targets on the ground by assuming zero target altitude, and improved the localization accuracy through expanded Kalman filtering. This method, however, applies to the localization only on a flat ground area. The above methods are more for near-distance ground targets and less for remote airborne targets. 

To deal with the above issues, this paper proposes an improved intersection localization method. Learning from the intersection measurement method of ground-based photoelectric theodolites [4], two optoelectronic platforms with angulation function are used simultaneously to measure the pitch and azimuth angles of the target in relation to platform coordinate system. Then the angle information is integrated with the information on UAV position and attitude to determine the azimuth/pitch angles of the LOS (line of sight, from the UAV to the target) in relation to terrestrial rectangular coordinates [23]. Finally, the target location can be obtained through intersection measurement. Independent of the movement style of targets, this system can locate airborne and ground-based moving objects in a real-time manner. Since the distance between the target and the platform is not needed in localization resolving, the installation of LRF in the optoelectronic platform is not necessary. This can effectively reduce the load on UAVs, escape from the LRF range restriction, and expand the localization applicability. 

## 2. Introduction of the Traditional Single-Station REA Localization Method

**Figure 1:**
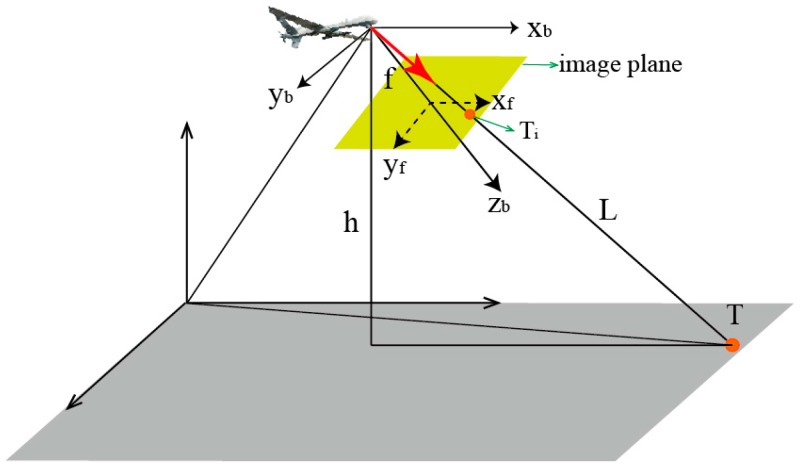
Schematic diagram of single-station localization.

Over the past decade the most popular algorithm used in airborne positioning is the single-station REA positioning method [9,16,17,22], which is shown in Figure 1. In Figure 1, *f* is the focal length, *T* represents the actual target point, and *T_i_* represents the position of the target point in the image whose coordinates in the image are expressed as (*u_x_*, *u_y_*). According to the method illustrated in [16], the three-dimensional coordinates of the target in the camera coordinate can be obtained:

(1) $$\left[\begin{array}{l} x_{t}\\ y_{t}\\ z_{t} \end{array}\right]=\frac{R}{F}\left[\begin{array}{l} u_{x}\\ u_{y}\\ f \end{array}\right]$$

where *R* is the distance from the camera to the target point, and *F* represents the distance from the origin of the camera coordinate to the target image point, $F=\sqrt{u_{x}^{2}+u_{y}^{2}+f^{2}}$ . The main problem of location is how to get the value of *R* with other known parameters. If the measured region is a flat ground surface, the value of *R* can be estimated by the method described in [22]. The distance between the camera and the ground plane *R* is estimated from the direction vector of LOS in the ground coordinate system. This method can effectively control the cost of the system and reduce the weight and volume of the platform. However, the precision of this algorithm is limited, thus it cannot meet the high-precision target location requirement in the UAV reconnaissance field. By using a LRF mounted inside the optoelectronic platform, which is used to achieve the distance between the measurement object and the platform, the value of *R* can also be obtained. Although this method is simple and direct, and has a high accuracy, adding an LRF in the platform will obviously increase the weight and volume of the photoelectric platform, which requires the load capacity of the UAV to be large enough.

This paper proposes a two-UAV intersection localization method to solve the problem of single-station positioning, which uses two devices locating the target at the same time to solve the problem of the localization that is needed to estimate the distance *R*. Compared with the single-station positioning algorithm, the two-UAV intersection localization algorithm has a higher positioning accuracy under the same measurement environment, which is verified in Section 5.3.

In summary, compared with the commonly used single-station localization method, the localization system proposed in this paper has higher location accuracy. Moreover, it can be applied in more types of UAVs since the platform used is lighter. However, it also has its own shortcomings, that is, more technical difficulty and higher cost of flight.

## 3. Makeup and Operating Principle of Two-UAV Intersection Localization System

**Figure 2:**
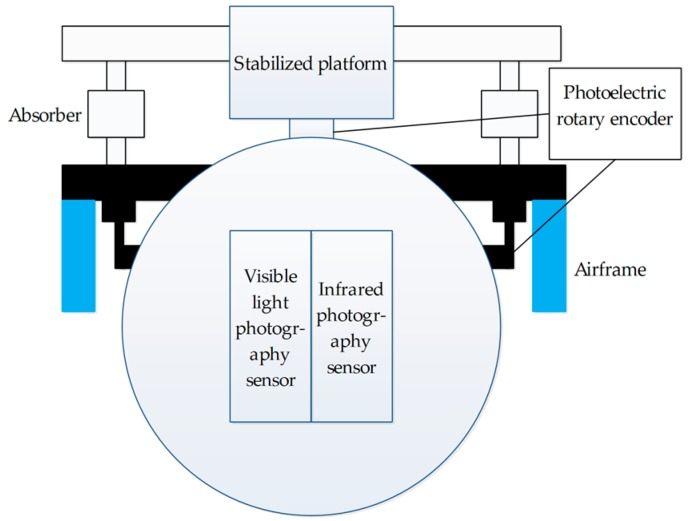
Structure chart of the onboard optoelectronic platform.

This system is mainly made up of two UAVs and their onboard optoelectronic platforms, GPS, and INS. As shown in the Figure 2, there are a stabilized platform, photoelectric rotary encoder, infrared photography sensor, and infrared photography sensor in the onboard optoelectronic platform. Where the photoelectric rotary encoders are used to measure the pitch angle and azimuth angle of the photography sensors, the photography sensors are used to obtain image data and provide information on the miss distance. The stabilized platform is made up of servo motors and gyroscopes, which can keep the photography sensors steady and control the platform to rotate according to the orders. The platform has two degrees of freedom, i.e., pitch and azimuth rotation, with the functions of reconnaissance, tracking, and localization.

**Figure 3:**
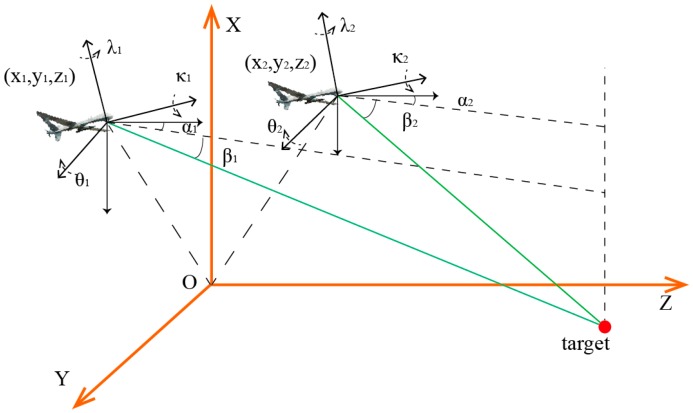
Schematic diagram of two-UAV intersection localization.

As shown in Figure 3, once the target is detected, the optoelectronic platform locks the target to the FOV (field of view) center and keeps tracking. During the tracking process, once the image target deviates from the center of vision due to the UAV and target relative position changes, the system can measure the miss distance (*u_x_*, *u_y_*) and transmit the miss distance to the control computer in real-time. According to the miss distance data, the platform servo control system can adjust the pitch and azimuth angles of the platform immediately to relock the target to the center of the FOV, in this case the target image has been ensured to be located in the center of the FOV. The optoelectronic platform locks the target to the FOV center and simultaneously measures the pitch and azimuth angles of LOS in relation to attitude measurement system. At the same time, the GPS/INS positioning system outputs the information on position and attitude of the two UAVs. Through the homogeneous-coordinates transformation, the output information is fused into a uniform coordinate system, where the target coordinates are solved through the intersection algorithm.

## 4. Key Technologies of Two-UAV Intersection Localization System

### 4.1. Establishment of Space Coordinates

**Figure 4:**
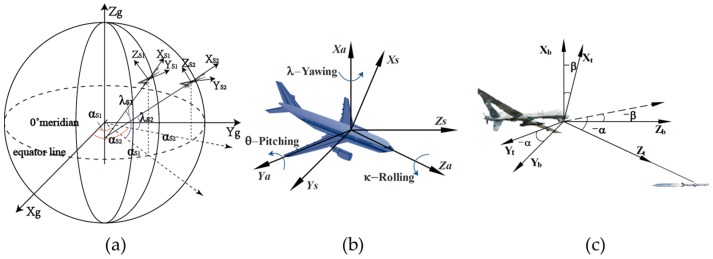
Definition of coordinate systems and their relations. (**a**) correlation diagram of terrestrial rectangular coordinate and geographic coordinate; (**b**) correlation diagram of geographic coordinateand UAV coordinate; (**c**) correlation diagram of UAV coordinate and camera coordinate.

The system described in this paper has six sets of space coordinates, as described below:

1. Geodetic coordinate system $C(\lambda_{s},\alpha_{s},h_{s})$ : based on international terrestrial reference system WGS-84. The position of any spatial point is expressed by longitude, latitude, and geodetic height $\left(\lambda_{s},\alpha_{s},h_{s}\right)$ [24].
2. Terrestrial rectangular coordinate system $G(O_{g}-X_{g}Y_{g}Z_{g})$ : an inertial coordinate system, as shown in the Figure 4a, where any spatial position is described by (x,y,z) . The origin is the center of Earth’s mass. The axis $Z_{g}$ points to the North Pole, and the axis $X_{g}$ is directed to the intersection point of the Greenwich meridian plane and equator. The axis $Y_{g}$ is normal to the plane $X_{g}O_{g}Z_{g}$ and constitutes, along with the axes $Z_{g}$ and $X_{g}$ , a Cartesian coordinate system.
3. Geographic coordinate system of UAV $S(O_{s}-X_{s}Y_{s}Z_{s})$ : as shown in the Figure 4a, the origin is the position $\left(\lambda_{s},\alpha_{s},h_{s}\right)$ of a UAV at a certain moment, the $Z_{s}$ points to true north, the $X_{s}$ points to zenith, and the $Y_{s}$ , along with $Z_{s}$ and $X_{s}$ , constitutes a right-handed coordinate system.
4. UAV coordinate system $A(O_{a}-X_{a}Y_{a}Z_{a})$ : as shown in the Figure 4b, the origin of this coordinate system coincides with that of UAV geographic coordinate system, the $X_{a}$ points to the direction right above the aircraft, the $Z_{a}$ points to the aircraft nose, and the $Y_{a}$ , along with $X_{a}$ and $Z_{a}$ , forms a right-handed coordinate system. The relationship between the UAV coordinate system and geographic coordinate system is shown in the Figure 4b. The tri-axial attitude angles are λ,θ,κ measured by the inertial navigation system.
5. Camera coordinate system $T(O_{t}-X_{t}Y_{t}Z_{t})$ : the origin is the intersection of the LOS and horizontal platform axis, and axis $Z_{t}$ is the telescope’s optic axis pointing to the target. When the axis $Z_{t}$ is in the initial (or horizontal) position, the axis $X_{t}$ will be directed to zenith, and the axis $Y_{t}$ , along with $Z_{t}$ and $X_{t}$ , will constitute a right-handed coordinate system. Figure 4c shows the relationship between camera coordinate system and UAV coordinate system.
6. Reference coordinate system $R(O_{r}-X_{r}Y_{r}Z_{r})$ : an auxiliary coordinate system built to facilitate intersection resolution. The origin is a definite point on the Earth ellipsoid, and the tri-axial directions are the same as in the Earth-rectangular coordinate system.

The geodetic coordinate system C can be converted into the terrestrial rectangular coordinate system G in accordance with the following equation:

(2) $$\{\begin{array}{l} x_{g}=(N+h_{s})\cos\alpha_{s}\cos\lambda_{s}\\ y_{g}=(N+h_{s})\cos\alpha_{s}\sin\lambda_{s}\\ z_{g}=[N(1-e^{2})+h_{s}]\sin\alpha_{s} \end{array}$$

The terrestrial rectangular coordinate system G can be converted into the geodetic coordinate system C in accordance with the following equation:

(3) $$\begin{array}{l} \lambda_{s}=a\tan(\frac{y_{g}}{x_{g}})\\ \alpha_{s}=a\tan(\frac{z_{g}+e_{1}^{2}b\sin^{3}\lambda_{s}}{\sqrt{x_{g}^{2}+y_{g}^{2}}-e^{2}a\cos^{3}\lambda_{s}})\\ h_{s}=\frac{\sqrt{x_{g}^{2}+y_{g}^{2}}}{\cos\alpha_{s}}-N \end{array}$$

where *a* is the length of the semi-major axis of the reference spheroid, and *b* is the length of semi-minor axis of the reference spheroid.

The first eccentricity: $e=\sqrt{\frac{a^{2}-b^{2}}{a^{2}}}$
The second eccentricity: $e_{1}=\sqrt{\frac{a^{2}-b^{2}}{b^{2}}}$
Radius of curvature in the prime vertical: $N=\frac{a}{\sqrt{1-e^{2}\sin^{2}\alpha_{s}}}$ .

As shown in the Figure 4c, the camera coordinate system T revolves around $Y_{t}$ for −β and around $X_{t}$ for −α to become the UAV coordinate system A in accordance with the principle of homogeneous-coordinates conversion:

(4) $$R_{t}^{a}(\alpha ,\beta )=R(\alpha )R(\beta )=\left[\begin{array}{cccc} C_{\beta } & 0 & S_{\beta } & 0\\ S_{\alpha }S_{\beta } & C_{\alpha } & -S_{\alpha }C_{\beta } & 0\\ -C_{\alpha }S_{\beta } & S_{\alpha } & C_{\alpha }C_{\beta } & 0\\ 0 & 0 & 0 & 1 \end{array}\right]$$

where $C_{\alpha }=\cos\alpha$ , $S_{\alpha }=\sin\alpha$ , and both *α* and *β* are the position angles in the UAV coordinate system. As shown in Figure 4b, the UAV coordinate system A revolves around $Z_{a}$ for κ , around $Y_{a}$ for θ , and around $X_{a}$ for λ to become the geographic coordinate system S:

(5) $$R_{a}^{S}(\lambda ,\theta ,\kappa )=R(\lambda )R(\theta )R(\kappa )=\left[\begin{array}{cccc} C_{\theta }C_{\kappa } & C_{\theta }S_{\kappa } & -S_{\theta } & 0\\ S_{\lambda }S_{\theta }C_{\kappa }-C_{\lambda }S_{\kappa } & S_{\lambda }S_{\theta }S_{\kappa }+C_{\lambda }C_{\kappa } & S_{\lambda }C_{\theta } & 0\\ C_{\lambda }S_{\theta }C_{\kappa }+S_{\lambda }S_{\kappa } & C_{\lambda }S_{\theta }S_{\kappa }-S_{\lambda }C_{\kappa } & C_{\lambda }C_{\theta } & 0\\ 0 & 0 & 0 & 1 \end{array}\right]$$

where λ,θ,κ are tri-axial attitude angles of UAV coordinate system in relation to geographic coordinate system [25]. 

As shown in Figure 4a, the geographic coordinate system S can be converted into the terrestrial rectangular coordinate system G through a shift of $h_{s}$ along the axis $X_{s}$ , a rotation of $\lambda_{s}$ around $Y_{s}$ , a rotation of $-\alpha_{s}$ around $Z_{s}$ , and a shift of $-Ne^{2}\sin\lambda_{s}$ along the axis $Z_{s}$ :

(6) $$R_{s}^{g}(h_{s},\lambda_{s},\alpha_{s},-Ne^{2}\sin\lambda_{s})=\left[\begin{array}{cccc} C_{\alpha_{S}}C_{\lambda_{S}} & -S_{\alpha_{S}} & -C_{\alpha_{S}}S_{\lambda_{S}} & h_{s}\\ S_{\alpha_{S}}C_{\lambda_{S}} & C_{\alpha_{S}} & -S_{\alpha_{S}}S_{\lambda_{S}} & 0\\ S_{\lambda_{S}} & 0 & C_{\lambda_{S}} & -Ne^{2}\sin\lambda_{s}\\ 0 & 0 & 0 & 1 \end{array}\right]$$

The terrestrial rectangular coordinate system G can be converted into the reference coordinate system R through a shift of $x_{r}$ along the axis $X_{g}$ , a shift of $y_{r}$ along the axis $Y_{g}$ , and a shift of $z_{r}$ along the axis $Z_{g}$ :

(7) $$R_{g}^{r}(x_{r},y_{r},v_{r})=\left[\begin{array}{cccc} 1 & 0 & 0 & x_{r}\\ 0 & 1 & 0 & y_{r}\\ 0 & 0 & 1 & z_{r}\\ 0 & 0 & 0 & 1 \end{array}\right]$$

### 4.2. Establishment of the Two-UAV Intersection Localization Model

**Figure 5:**
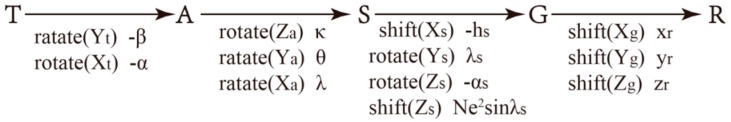
Coordinate transformation process.

As shown in Figure 4c, when tracking a target, the airborne optoelectronic platform will adjust the camera angle and lock the target to the FOV center. At this moment, both the LOS and the axis $Z_{t}$ of the camera coordinate system are directed to the target. In the case of localization, the two UAVs will output and convert the measured data simultaneously into a uniform coordinate system for processing. In the camera coordinate system, the unit vector of LOS is expressed as ***L****_i_* = [0, 0, *f*, 1]^T^, where *f* is camera focus. Through the coordinate transformation, the expression of the LOS vector in the reference coordinate system can be obtained. Then the expression of the target position can be determined through the intersection algorithm. The coordinate transformation process is shown in the Figure 5.

Where *α* and *β* are the azimuth and pitch angles of camera in relation to UAV and can be measured by a photoelectric encoder; λ,θ,κ are the attitude angles of UAV in relation to geographic coordinate system and can be measured by the inertial navigation system; $\left(\lambda_{s},\alpha_{s},h_{s}\right)$ can be measured by GPS; *N* is the radius of curvature in the prime vertical; *e* is eccentricity; and the coordinates (*x_r_*, *y_r_*, *z_r_*) are the expression of reference coordinate system in the terrestrial rectangular coordinate system. The data $\left(L,B,H)=(\lambda_{s},\alpha_{s},h_{s}\right)$ measured by GPS are the coordinates in the geodetic coordinate system. However, in the actual localization resolution, they shall be converted into the coordinates in the reference coordinate system for ease of calculation by determining, at first, their values in the terrestrial rectangular coordinate system and then converting these values into the coordinates in the reference coordinate system through the transformation process shown in Figure 5. 

For the convenience of expression, the parameters measured by the two UAVs are marked by *i* (*i* = 1, 2). Through the process in Figure 5, the value of the LOS vector ***L****_gi_* in the reference coordinate system can be determined:

(8) $$L_{gi}=\left(\begin{array}{l} l_{gi}\\ m_{gi}\\ n_{gi}\\ 1 \end{array}\right)=R_{g}^{r}R_{s}^{g}R_{a}^{s}R_{t}^{a}L_{i}$$

**Figure 6:**
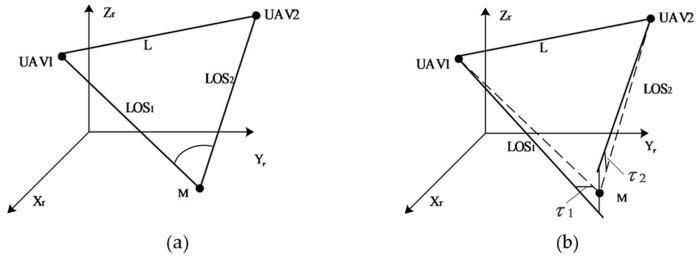
Positional relationship of the two visual axes. (**a**) rendezvous; (**b**) non-uniplannar intersection.

In fact, there exist some measurement errors in the positioning calculation process leading to the deviation between the solution of the visual axis vector direction and the actual measurement of the visual axis. Thus, the visual axis LOS1, LOS2 in the reference coordinate system may be rendezvous (shown in Figure 6a) or non-uniplannar intersections (shown in Figure 6b). As shown in Figure 6b, the dotted lines represent the actual visual axis, the solid lines represent the visual axis obtained from calculating Equation (8) and $\tau_{1},\tau_{2}$ express the deviations of the dotted lines and the solid lines. In order to solve the problem of locating the target, we introduce a point M $\left(x_{m},y_{m},z_{m}\right)$ in the space according to the estimated target position, and the distance of M to the two visual axes LOS1, LOS2 is a minimum based on the spatial straight line principle.

*E* $\left(x_{m},y_{m},z_{m}\right)$ can be calculated according to spatial geometry knowledge:

(9) $$E\left(x_{m},y_{m},z_{m}\right)=\sqrt{\sum_{i=1}^{2}\left[{\left(x_{m}-x_{i}^{F}\right)}^{2}+{\left(y_{m}-y_{i}^{F}\right)}^{2}+{\left(z_{m}-z_{i}^{F}\right)}^{2}\right]}$$

where the coordinate $\left(x_{i},y_{i},z_{i}\right)$ shows the position of the UAV in the reference coordinate system at a certain time and $\left(x_{i}^{F},y_{i}^{F},z_{i}^{F}\right)$ are the foot coordinates of the pedal of the point M to LOSi, which can be obtained according to the linear parameter equation:

(10) $$\begin{array}{l} x_{i}^{F}=x_{i}+l_{gi}\left[l_{gi}\left(x_{m}-x_{i}\right)+m_{gi}\left(y_{m}-y_{i}\right)+n_{gi}\left(z_{m}-z_{i}\right)\right]\\ y_{i}^{F}=y_{i}+m_{gi}\left[l_{gi}\left(x_{m}-x_{i}\right)+m_{gi}\left(y_{m}-y_{i}\right)+n_{gi}\left(z_{m}-z_{i}\right)\right]\\ z_{i}^{F}=z_{i}+n_{gi}\left[l_{gi}\left(x_{m}-x_{i}\right)+m_{gi}\left(y_{m}-y_{i}\right)+n_{gi}\left(z_{m}-z_{i}\right)\right] \end{array}$$

In this case, the problem of the two-UAV intersection localization can be simplified to find out the coordinates $\left(x_{m},y_{m},z_{m}\right)$ to make the value of *E* the smallest. According to the principle of least squares, the partial derivatives of *x_m_*, *y_m_*, *z_m_* for *E* can be found and assigned to 0:

(11) $$\begin{array}{l} \sum_{i=1}^{2}\left[\left(1-l_{gi}^{2}\right)\left(x_{m}-x_{i}\right)-l_{gi}m_{gi}\left(y_{m}-y_{i}\right)-l_{gi}n_{ni}\left(z_{m}-z_{i}\right)\right]=0\\ \sum_{i=1}^{2}\left[-l_{gi}m_{gi}\left(x_{m}-x_{i}\right)+\left(1-m_{gi}^{2}\right)\left(y_{m}-y_{i}\right)-m_{gi}n_{ni}\left(z_{m}-z_{i}\right)\right]=0\\ \sum_{i=1}^{2}\left[-l_{gi}n_{gi}\left(x_{m}-x_{i}\right)+m_{gi}n_{ni}\left(y_{m}-y_{i}\right)+\left(1-n_{gi}^{2}\right)\left(z_{m}-z_{i}\right)\right]=0 \end{array}$$

According to the formula, the linear equations of *x_m_*, *y_m_*, *z_m_* can be rewritten in the form of matrix AM=b , where:

$$A=\left(\begin{array}{ccc} \sum_{i=1}^{2}\left(1-l_{gi}^{2}\right) & -\sum_{i=1}^{2}l_{gi}m_{gi} & -\sum_{i=1}^{2}l_{gi}n_{gi}\\ -\sum_{i=1}^{2}l_{gi}m_{gi} & \sum_{i=1}^{2}\left(1-m_{gi}^{2}\right) & -\sum_{i=1}^{2}m_{gi}n_{gi}\\ -\sum_{i=1}^{2}l_{gi}n_{gi} & -\sum_{i=1}^{2}m_{gi}n_{gi} & \sum_{i=1}^{2}\left(1-n_{gi}^{2}\right) \end{array}\right),\;M=\left(\begin{array}{l} x_{m}\\ y_{m}\\ z_{m} \end{array}\right),\;b=\left(\begin{array}{c} \sum_{i=1}^{2}\left[\left(1-l_{gi}^{2}\right)x_{i}-l_{gi}m_{gi}y_{i}-l_{gi}n_{ni}z_{i}\right]\\ \sum_{i=1}^{2}\left[-l_{gi}m_{gi}x_{i}+\left(1-m_{gi}^{2}\right)y_{i}-m_{gi}n_{ni}z_{i}\right]\\ \sum_{i=1}^{2}\left[-l_{gi}n_{gi}x_{i}-m_{gi}n_{ni}y_{i}+\left(1-n_{gi}^{2}\right)z_{i}\right] \end{array}\right)$$

Since *A* is a nonsingular matrix, the solution of the system of linear equations can be obtained: $M=A^{-1}b$ . Through the homogeneous-coordinates transformation expression $R_{r}^{g}$ , the coordinates of the target in the terrestrial rectangular coordinate system can be obtained. From Equation (3), the coordinates (*B_m_*, *L_m_*, *H_m_*) of the target *M* in the geodetic coordinate system can be derived.

## 5. Accuracy Analysis and Simulation Experiment

Localization accuracy analysis is an important step to judge whether a localization algorithm is good or not. There are mainly two factors influencing the accuracy of a localization algorithm. The first factor is the error of a measurement parameter. It can be learned from Equation (8) that various parameters will be integrated into the solution process of the target and their errors will undoubtedly influence the final localization accuracy. The second factor is the measuring position of a UAV in relation to the target, which, during the intersection measurement, has an important influence on measurement accuracy. In reality, the measurement accuracy can only reach a certain level due to limited modern technological and design development and equipment production costs. In this case, the positional relationship between the UAV and the target is important to localization accuracy.

### 5.1. Influence of UAV Position on Localization Accuracy

UAV position is of great implication to localization accuracy. When locating a target during actual military reconnaissance, the UAV needs to keep enough distance from the target to ensure stealthiness. In view of the above background, this paper analyzed the influence of different measurement positions on localization accuracy from the following aspects and obtained the relevant data for engineering reference. The measurement errors of various sensors used in the test are determined according to the maximum nominal errors given by the equipment specifications. This paper assumes that the same measuring equipment are adopted by the two UAVs, which means the parameter errors are governed by the same criteria, as shown in the Table 1.

The simulation described in this paper is carried out in the reference coordinate system, where the x axis indicates altitude and the YOZ plane represents the horizontal plane. Since the following simulation test is mainly to demonstrate the influence of UAV position in relation to the target on localization accuracy, the UAV attitude angle will not affect the test results. To facilitate the observation of test results, the three axes of the UAV coordinate system in the test are parallel to those of the reference coordinate system.

#### 5.1.1. Influence of Baseline Length on Localization Accuracy

**Figure 7:**
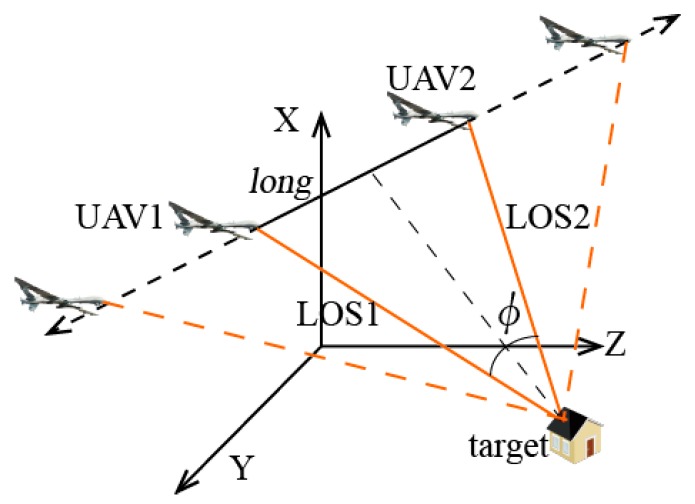
Positional relationship between UAVs and the target.

The target is tracked by two UAVs at the same distance (namely, the two UAVs and the target form an isosceles triangle), as shown in the Figure 7.

**Figure 8:**
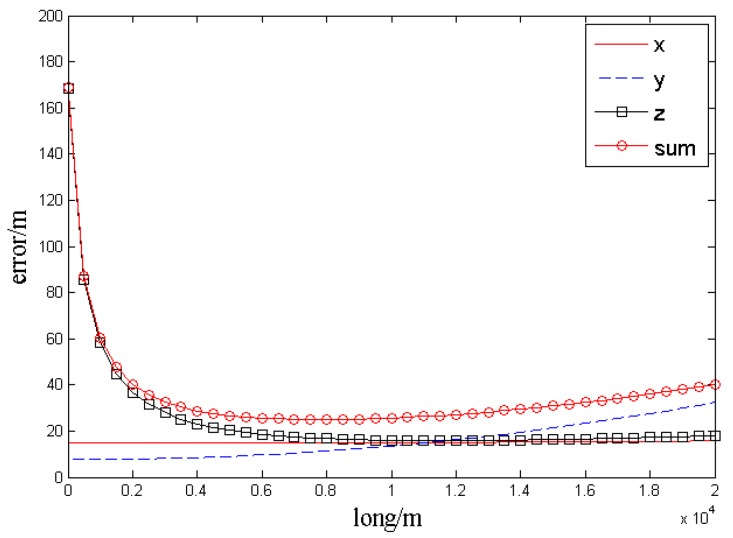
Error curves.

The distances between the target and UAV baselines are 5 km and remain unchanged. By changing the baseline *long*, the analysis result is obtained, as shown in the Figure 8.

In the Figure 8, the curves *x*, *y*, and *z* represent the localization errors Δx,Δy,Δz ( root mean square (RMS) errors rooted in 100 simulation times at one point) in the three axes. The curve *sum* indicates the error radius of the actual target position and measurement point, namely $sum=\sqrt{\Delta x^{2}+\Delta y^{2}+\Delta z^{2}}$ . The test results show that, when the target is tracked by two UAVs at the same distance and altitude, and the distances from the target to UAV baselines are set as 5 km, if *long* = 7.35 km, the intersection angle ϕ will be 72.6318° and the localization accuracy will be the highest: Δx=14.83 m , Δy=11.31 m , Δz=16.68 m , *sum* (min) = 25.0235 m. When the baseline *long* is 2.5–16.3 km, the intersection angle will vary from 28.1° to 116.8°, and both the total error and every error component will be 32 m. The localization result should be rejected because of the excessive error once the intersection angle is out of the range.

#### 5.1.2. Localization Accuracy of Two Tracking UAVs at the Same Altitudes but Different Distances with the Target

**Figure 9:**
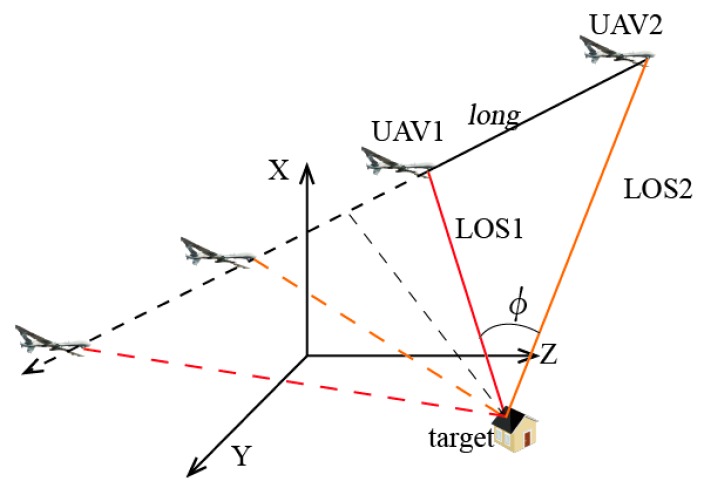
Positional relationship between the UAVs and the target.

It is known from the above that, when the distances from the target to UAV baselines are still kept as 5 km and the baseline *long* is 7.35 km, the localization accuracy will be the highest. Under these conditions, the two simulated UAVs move along the baseline extension, as shown in the Figure 9.

**Figure 10:**
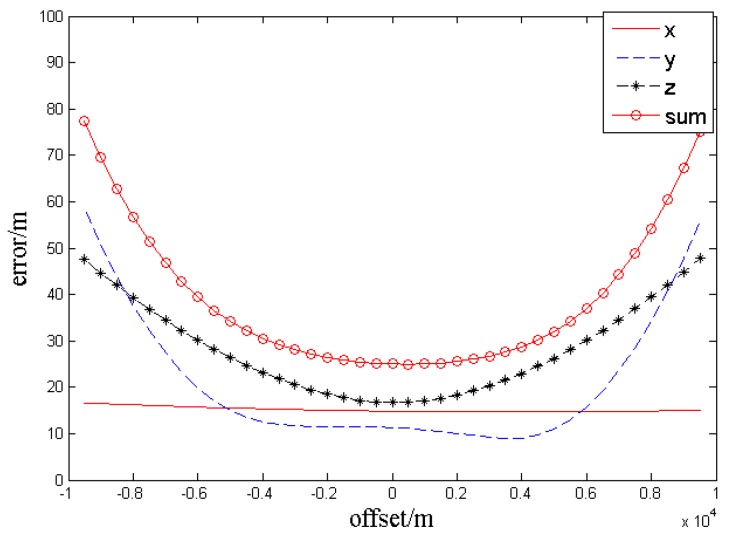
Error curves.

As shown in the Figure 9, when the coordinates of simulated target are (0, 3675, 4000) and UAV 1 moves linearly at a constant speed from (3000, −10,000, 0) to (3000, 10,000, 0) and UAV 2 from (3000, −2650, 0) to (3000, 17,350, 0) at the same speed, the localization accuracy will be determined as shown in the Figure 10. 

The simulation results show that the localization error increases with the *offset*. When the offset is 0 (the distances between the two UAVs and the target are the same), the total localization error (*sum*) is 25.0235 m, or the minimum. When the *offset* is 2.5 km, the *sum* is 27.1464 m. When the *offset* is 5 km, the *sum* is 34.1459 m. Once the *offset* exceeds 5 km, the localization result should be rejected because of the excessive error.

From the above simulation information, the following conclusions can be drawn:

1. According to the localization algorithm proposed by this paper, the optimal position for the two-UAV intersection system to locate the target is when the two UAVs and the target are on the same horizontal line and the azimuth of UAV 1 in relation to the target is just the opposite of that of UAV 2, namely the positions of the two UAVs in relation to the target are the same, with an intersection angle of 72.6318°. In this paper, the distance from the simulated target to a UAV baseline is 5 km and, accordingly, the baseline length in the optimal measuring position is *long* = 7.35 km. In this case, the *x*-axis error Δx is 14.83 m, the *y*-axis error Δy is 11.31 m, the *z*-axis error Δz is 16.68 m, and the error radius *sum* is 25.0235 m.
2. In the real world, both the target and the UAVs are moving continuously along unpredictable tracks, so it is very difficult for the UAVs to remain in the optimal measuring positions for target localization. When the two UAVs are kept parallel to each other and at the same horizontal plane as the target, an intersection angle of 28.1°–116.8° can achieve a desirable localization result.

### 5.2. Modified Adaptive Kalman Filtering during Two-UAV Intersection Localization

To improve the localization accuracy, the observations for the target shall be filtered. The target aimed by our system is moving in the air or on the ground. A dynamic localization model can be established by adopting the modified adaptive Kalman filtering algorithm and taking the target’s triaxial movement positions and speeds as state variables, and its target position as measurement variables. In this paper, the modified Saga adaptive filtering method is used to solve the problem that the statistical characteristics of system noise and observation noise of the dynamical target are uncertain. The basic process is to calculate the system noise at the current time and the estimated value of observation noise, and compute the state estimation values by employing the new information based on each measured value ***Y***(*k*).

#### 5.2.1. Modified Adaptive Kalman Filtering Modeling

An airborne optoelectronic platform often locates the targets thousands of meters away. Considering that both the target speed and the UAV speed-to-altitude ratio are not large, the target motion in the tri-axial directions can be approximated as uniform motion. Suppose the sampling time is T. The state equation of target motion will be:

(12) X(k)=AX(k−1)+BU(k−1)+W(k−1)

where X(k) is the target state variable at the time *k*; $X(k)=[x(k),v_{x}(k),y(k),v_{y}(k),z(k),v_{z}(k)]$ ^T^, where $x(k),v_{x}(k),y(k),v_{y}(k),z(k),v_{z}(k)$ are the target’s positions and speeds on the three axes. The system has no controlled variables. If ***B***(*k*) = 0, the state-transition matrix will be:

$$A=\left[\begin{array}{cccccc} 1 & T & 0 & 0 & 0 & 0\\ 0 & 1 & 0 & 0 & 0 & 0\\ 0 & 0 & 1 & T & 0 & 0\\ 0 & 0 & 0 & 1 & 0 & 0\\ 0 & 0 & 0 & 0 & 1 & T\\ 0 & 0 & 0 & 0 & 0 & 1 \end{array}\right]$$

***W***(*k*) is a white Gaussian noise sequence for system state noise, with the expectation of ***q***(*k*) and the covariance of ***Q***(*k*).

The system measurement equation is as follows:

(13) Y(k)=HX(k)+V(k)

where *Y*(*k*) is the system measurement, ***Y***(*k*) = [*y*_x_(*k*), *y*_y_(*k*), *y*_z_(*k*)]^T^, ***H*** is system observation matrix,

$$H=\left[\begin{array}{cccccc} 1 & 0 & 0 & 0 & 0 & 0\\ 0 & 0 & 0 & 0 & 0 & 0\\ 0 & 0 & 1 & 0 & 0 & 0\\ 0 & 0 & 0 & 0 & 0 & 0\\ 0 & 0 & 0 & 0 & 1 & 0\\ 0 & 0 & 0 & 0 & 0 & 0 \end{array}\right]$$

***V***(*k*) is a white Gaussian noise sequence for observation noise, with the expectation of ***v***(*k*) and the covariance of ***R***(*k*).

The recurrence equation of adaptive Kalman filter can be obtained:

(14) X(k/(k−1))=A(k)X(k−1)+q(k−1)

(15) $$P(k/(k-1))=A(k)P(k-1)A^{T}(k)+Q$$

(16) $$K(k)=\frac{P(k/(k-1))H^{T}(k)}{\left(H(k)P(k/(k-1))H^{T}(k)+R(k)\right)}$$

(17) X(k)=X(k/k−1)+K(k)(Y(k)−HX(k/k−1)−v(k−1))

(18) P(k)=(1−K(k)H(k))P(k/(k−1))

This paper uses a considerable number of error arithmetic mean values to approximate the mathematical expectation of the errors, and then uses these errors and mathematical expectations to estimate the variance of the errors:

(19) $$\begin{array}{l} q\left(k\right)=\frac{1}{k}\sum_{j=1}^{k}\left(X(k)-A(k)X(k-1)\right)\\ q_{w}\left(k\right)=\frac{1}{k}\sum_{j=1}^{k}\left(X(k)-A(k)X(k-1)-q(k)\right)\\ r\left(k\right)=\frac{1}{k}\sum_{j=1}^{k}\left(X(k)-H(k)X(k/k-1)\right)\\ r_{w}\left(k\right)=\frac{1}{k}\sum_{j=1}^{k}\left(X(k)-H(k)X(k/k-1)-r(k)\right) \end{array}$$

This estimation method is an unbiased estimate in which ***q***_w_(*k*), ***r***_w_(*k*) represent the standard deviation of the estimate. The square of the elements in ***q***_w_(*k*) are taken as the diagonal elements of ***Q***(*k*), the other elements of ***Q***(*k*) are assigned 0, the square of the elements in ***r***_w_(*k*) are taken as the diagonal elements of ***R***(*k*), and the other elements of ***R***(*k*) are assigned 0. The noise statistics are obtained in this way. For the time-varying system, the noise changes with time, and the old data needs to be removed, so this article uses 100 before the current time of the unbiased data, which can guarantee the accuracy and real-time of the data.

#### 5.2.2. Filter Initialization

An adaptive Kalman filtering algorithm is a recurrence algorithm and, thus, must be initialized. This paper uses the optimal localization position for testing, so the observation noise ***V***(*k*) is a white Gaussian noise whose covariance ***v***(*k*) is a constant, namely ***R***(1) = diag(*r*1, *r*2, *r*3) = [14.83^2^, 0, 0; 0, 11.31^2^, 0; 0, 0, 16.68^2^]. The initial state variable ***X***(1) can be obtained from initial observations. Suppose the target moves at a constant speed in the tri-axial directions, then ***X***(1) = [*y*_x_(1), (*y*_x_(2) − *y*_x_(1))/*T*, *y*_y_(1), (*y*_y_(2) − *y*_y_(1))/*T*, *y*_z_(1), (*y*_z_(2) − *y*_z_(1))/*T*]. The initial covariance matrix can be written as:

$$P(1)=\left[\begin{array}{cccccc} r11 & \frac{r11}{T} & 0 & 0 & 0 & 0\\ \frac{r11}{T} & \frac{2r11}{T^{2}} & 0 & 0 & 0 & 0\\ 0 & 0 & r22 & \frac{r22}{T} & 0 & 0\\ 0 & 0 & \frac{r22}{T} & \frac{2r22}{T^{2}} & 0 & 0\\ 0 & 0 & 0 & 0 & r33 & \frac{r11}{T}\\ 0 & 0 & 0 & 0 & \frac{r11}{T} & \frac{2r33}{T^{2}} \end{array}\right]$$

The sampling time is *T* = 1 s. The filter starts to work when *k* = 2. 

#### 5.2.3. Test Results

**Figure 11:**
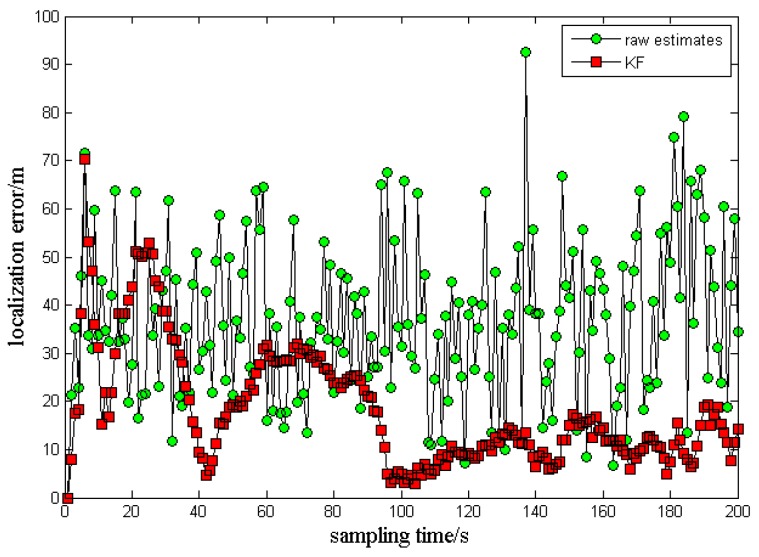
Localization errors after modified adaptive Kalman filtering.

**Figure 12:**
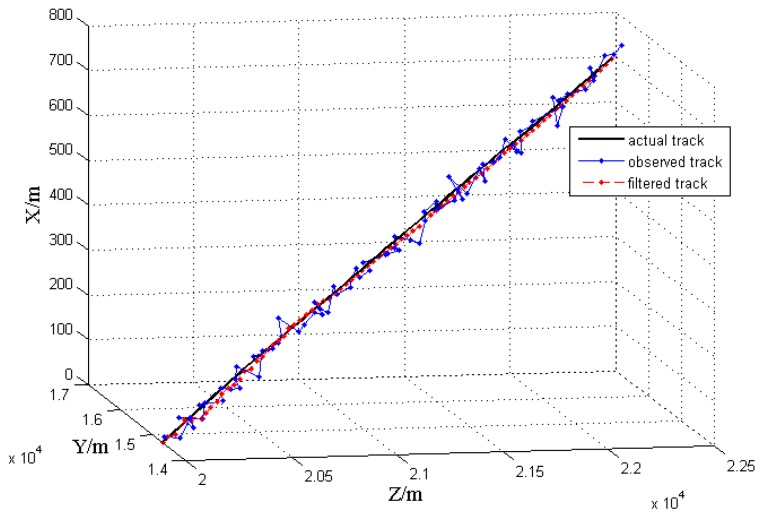
Localization track.

The filtering results from the above method are shown in the Figure 11 and Figure 12.

It is observed from the error distribution in Figure 11 that, after modified adaptive Kalman filtering, the localization error becomes 15.7983 m, much smaller than the original measurement of 25.0235 m. Thus, the localization accuracy has been improved significantly. Judging from the sampling time, the first 100 s constitute a data accumulation course, so the localization results are relatively divergent. However, after 100 s, the data begin to converge rapidly, and the error values are smoother than before filtering.

The Figure 12 shows the track curves of the simulated target in motion. Among them, the black curve is the actual track, the blue curve with solid dots is the observed track, and the red dashed curve is the filtered track. It can be seen that, the observed track is divergent, whereas the filtered track is smoother and fits the actual track better. 

### 5.3. Flight Data Results

In this section, the approach previously presented is now validated using flight test data. A ground receiving station is arranged to receive the measurement data and video sequences from the two UAVs and optoelectronic platforms, and the target position is calculated in real-time using the received data. In order to ensure each set of images and the parameters from the two UAVs that the ground receiving station used in the calculation process are acquired at the same time, time synchronization calibration for the two unmanned aerial vehicles and the photoelectric platforms is necessary to be taken prior to flight. The corresponding shooting time and serial number information of the image of each frame of the video captured should be noted in order to avoid framing errors in the solution. By using these methods, we can ensure that the image data and parameters used during the resolution are captured at the same time by the two UAVs. The video images are shot by a CMOS photo-detector with the resolution ratio 1024 × 768, a pixel size of 5.5 µm, and a frame rate of 25 frames per second. The clock error after the time calibration of two UAVs is 5 ms, so the time difference of arrival of the two images and measured parameters used in the solution process is up to 45 ms. For non-high-speed moving objects, the error is within the acceptable range. The terrestrial solution unit uses the high-speed DSP chip TMS320F28335 (made by the TI (Texas Instruments) company, Dallas, TX, USA) to receive the data and calculate the target position with processing time of 500 µs, which can meet the real-time requirements.

In order to verify the feasibility of the algorithm, the flight test is performed in an outfield environment. The two UAVs fly with a steady rate of 100–110 m/s, flight height of 5400 m, and the camera has a focal length of 200 mm. An outfield experiment of positioning a fixed building with the algorithm of two-UAV intersection localization is carried out. The exact positioning of the target is known. With the UAVs’ flight, the positional relationship between the UAVs and target is in flux. In this section several feature positions were selected to measure the localization error used to verify the simulation experiment.

**Figure 13:**
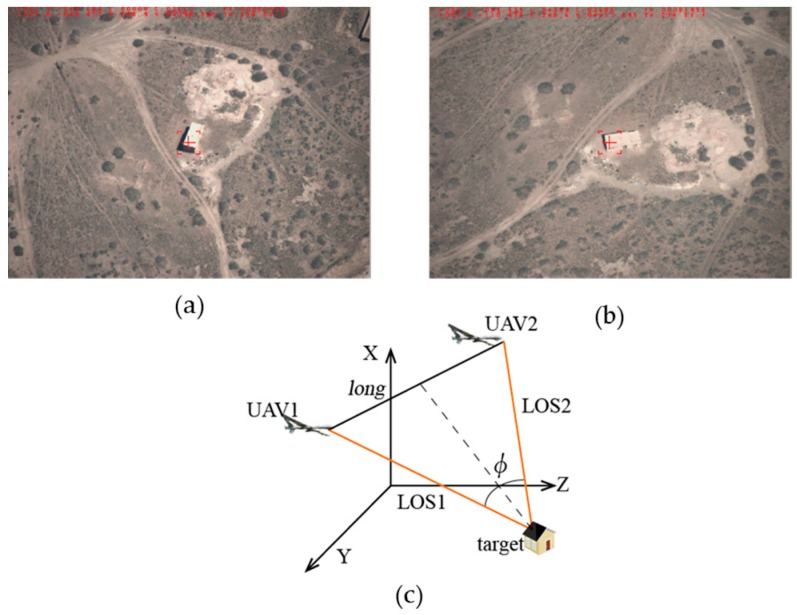
Feature position 1: (**a**) target detection by UAV1; (**b**) target detection by UAV2; and (**c**) positional relationship graph between the two UAVs and the target.

**Feature Position 1:** As shown in Figure 13a,b, the two images’ rooted in the outfield experimental video data are taken at the same time and their sizes are 1024 × 768 pixels. The positional relationship graph between the two UAVs and the target can be described as in Figure 13c, that is, the target is tracked by two UAVs at the same distance with the baselines *long =* 7332 m and the intersection angle ϕ 73.2°. In this case the localization error can be: $\sigma_{B}=1.63\times 10^{-4}\;({}^{\circ})$ , $\sigma_{L}=1.35\times 10^{-4}\;({}^{\circ})$ , $\sigma_{H}=15.8\;(m)$ , $\sigma_{sum}=27.6\;(m)$ , where $\sigma_{B}$ represents the longitude error, $\sigma_{L}$ represents the latitude error, $\sigma_{H}$ represents the altitude error, and $\sigma_{sum}$ represents the error radius.

**Figure 14:**
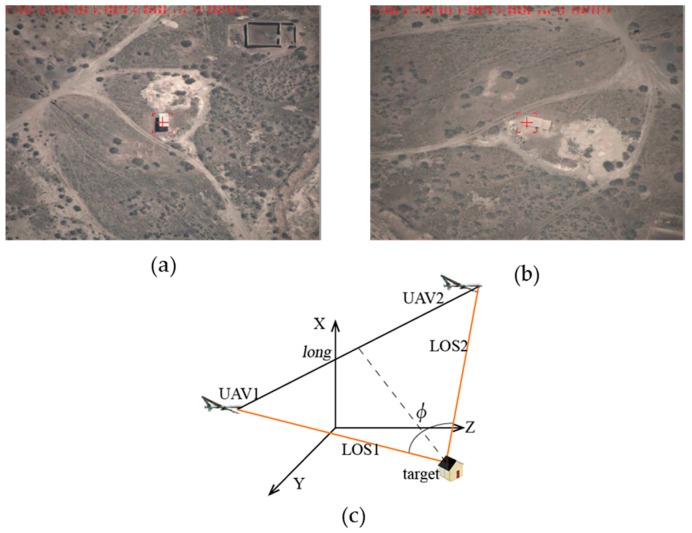
Feature position 2: (**a**) target detection by UAV1; (**b**) target detection by UAV2; and (**c**) positional relationship graph between the two UAVs and the target.

**Feature Position 2:** The positional relationship graph between the two UAVs and the target can be described as in Figure 14c, that is, the target is tracked by two UAVs at the same distance with the baselines *long =* 9337 m and the intersection angle ϕ 101.3°. In this case the localization error can be: $\sigma_{B}=2.83\times 10^{-4}\;({}^{\circ})$ , $\sigma_{L}=2.71\times 10^{-4}\;({}^{\circ})$ , $\sigma_{H}=21.3\;(m)$ , $\sigma_{sum}=40.6\;(m)$ .

**Figure 15:**
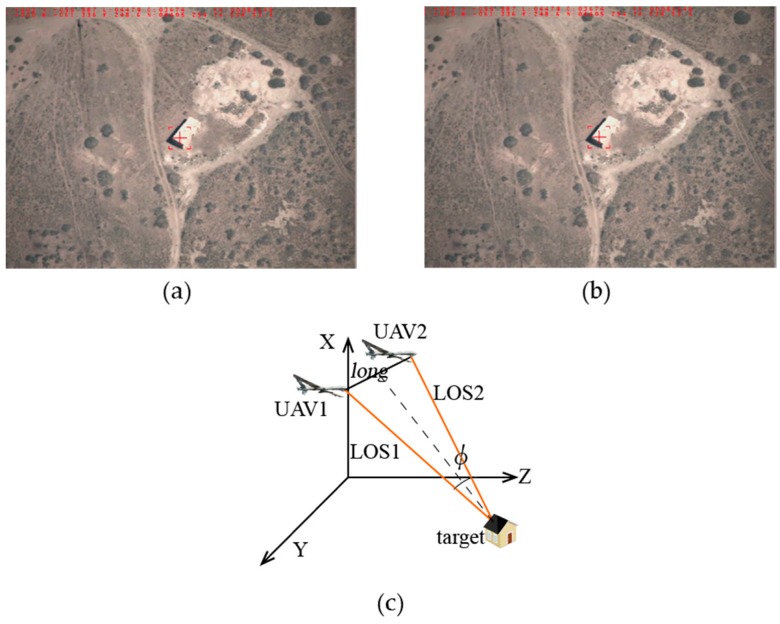
Feature position 3: (**a**) target detection by UAV1; (**b**) target detection by UAV2; and (**c**) positional relationship graph between the two UAVs and the target.

**Feature Position 3:** The positional relationship graph between the two UAVs and the target can be described as in Figure 15c, that is, the target is tracked by two UAVs at the same distance with the baselines *long =* 2749 m and the intersection angle ϕ 24.8°. In this case the localization error can be: $\sigma_{B}=1.45\times 10^{-3}\;({}^{\circ})$ , $\sigma_{L}=9.46\times 10^{-4}\;({}^{\circ})$ , $\sigma_{H}=118.6\;(m)$ , $\sigma_{sum}=198.3\;(m)$ .

**Figure 16:**
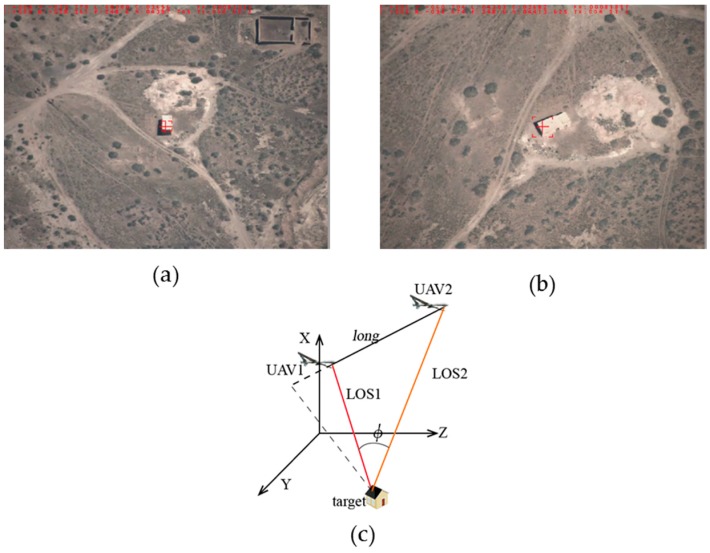
Feature position 4: (**a**) target detection by UAV1; (**b**) target detection by UAV2: and (**c**) positional relationship graph between the two UAVs and the target.

**Feature Position 4:** The positional relationship graph between the two UAVs and the target can be described as in Figure 16c, that is, the target is tracked by two UAVs at the same distance with the baselines *long =* 7332 m and the *offset =* 4200 m. In this case the localization error can be: $\sigma_{B}=2.38\times 10^{-4}\;({}^{\circ})$ , $\sigma_{L}=1.58\times 10^{-4}\;({}^{\circ})$ , $\sigma_{H}=19.8\;(m)$ , $\sigma_{sum}=36.2\;(m)$ .

As shown in Table 2, the results of flight experiments show that the positioning accuracy of the two-UAV intersection localization system is highest when the two UAVs and the target are at the same distance, and the localization precision is related to the intersection angle ϕ , which is basically the same as the simulation result. The actual localization accuracy is lower than the simulation result because there exist some unknown factors in the outfield actual flight test, and future work is to take them into consideration. The experiment proves the feasibility of the system and the accuracy of the simulation analysis.

In order to compare the positioning accuracy of this algorithm with the traditional single-station REA localization manner, the single-station localization experiment is added in this section. The experimental conditions are the same as above. The laser range-finder is installed inside the photoelectric platform. With laser ranging accuracy of 5 m and other equipment errors the same as the experiment before, the comparison results are shown in Table 2. When the positional relationship graph between the two UAVs and the target meets the requirements of the simulation results, the accuracy of the proposed method is obviously higher than that of the traditional stand-alone positioning method. On the contrary, as shown in Feature Position 3, when the intersection angle ϕ is beyond the 28.1°–116.8° scope of requirement, the accuracy of the proposed method is lower than that of the traditional stand-alone positioning method.

## 6. Conclusions

To address the limitation of the existing airborne optoelectronic localization method, this paper proposes an improved two-UAV intersection localization algorithm based on the conventional ground intersection localization method. This paper establishes a two-UAV intersection localization model, studies, in detail, the influence of UAV position on localization accuracy in order to find the optimal localization position, and quantifies the localization accuracy in different positions, thus providing a basis for the planning of the UAV track during localization. When the target is 5 km away from UAV baselines, the localization accuracy in the optimal localization position can reach 25.0235 m. For a target whose track is quite smooth, this paper introduces a modified adaptive Kalman filtering method to improve the localization accuracy to 15.7983 m. Finally, an outfield experiment was carried out to validate the two-UAV intersection localization algorithm. The localization accuracy in the optimal localization positions: $\sigma_{B}=1.63\times 10^{-4}\;({}^{\circ})$ , $\sigma_{L}=1.35\times 10^{-4}\;({}^{\circ})$ , $\sigma_{H}=15.8\;(m)$ , $\sigma_{sum}=27.6\;(m)$ , which is basically the same as the simulation result. Next, we will continue to study how to plan the UAV track and build a more accurate Kalman filtering model. 

## Figures and Tables

**Table 1:** Distribution of random errors.

Name of Error Variable	Random Distribution	Error *σ*
Miss distance *x*	Normal distribution	4.8 × 10^−5^ (m)
Miss distance *y*	Normal distribution	4.8 × 10^−5^ (m)
UAV longitude	Normal distribution	1 × 10^−4^ (°)
UAV latitude	Normal distribution	1 × 10^−4^ (°)
UAV altitude	Normal distribution	10 (m)
UAV pitch	Normal distribution	0.01 (°)
UAV roll	Normal distribution	0.01 (°)
UAV yaw	Normal distribution	0.05 (°)
Camera pitch	Uniform distribution	0.01 (°)
Camera azimuth	Uniform distribution	0.01 (°)

**Table 2:** Localization error.

Positional Relationship	Localization Algorithm	$\sigma_{B}$ (°)	$\sigma_{L}$ (°)	$\sigma_{H}$ (m)	$\sigma_{sum}$ (m)
Feature Position 1	Two-UAV localization	$1.63\times 10^{-4}$	$1.35\times 10^{-4}$	15.8	27.6
	single-station localization	$2.69\times 10^{-4}$	$1.51\times 10^{-4}$	19.4	37.4
Feature Position 2	Two-UAV localization	$2.83\times 10^{-4}$	$2.71\times 10^{-4}$	21.3	40.6
	single-station localization	$3.81\times 10^{-4}$	$4.23\times 10^{-4}$	45.2	72.8
Feature Position 3	Two-UAV localization	$1.45\times 10^{-3}$	$9.46\times 10^{-4}$	118.6	198.3
	single-station localization	$2.71\times 10^{-4}$	$1.84\times 10^{-4}$	21.3	38.5
Feature Position 4	Two-UAV localization	$2.38\times 10^{-4}$	$1.58\times 10^{-4}$	19.8	36.2
	single-station localization	$2.46\times 10^{-4}$	$3.35\times 10^{-4}$	24.6	51.8
